# OCTA Biomarkers Underlying Structure–Function Correlations in Idiopathic Epiretinal Membrane: A Systematic Review

**DOI:** 10.3390/diagnostics15202596

**Published:** 2025-10-15

**Authors:** Anca Mădălina Sere, George Adrian Muntean, Andreea Petra Cristea, Simona Delia Nicoară

**Affiliations:** 1 Doctoral School of Medicine, “Iuliu Hațieganu” University of Medicine and Pharmacy, 400023 Cluj-Napoca, Romania; andreeapetra30@yahoo.com; 2 Department of Ophthalmology, “Iuliu Hațieganu” University of Medicine and Pharmacy, 400012 Cluj-Napoca, Romania; simonanicoara1@gmail.com; 3 Medfuture Institute for Biomedical Research, “Iuliu Hațieganu” University of Medicine and Pharmacy, 400347 Cluj-Napoca, Romania; georgemuntean99@gmail.com; 4 Department of Ophthalmology, Emergency County Hospital, 400006 Cluj-Napoca, Romania

**Keywords:** idiopathic epiretinal membrane, optical coherence tomography angiography, functional outcome, structural biomarkers, pars plana vitrectomy

## Abstract

**Background:** Idiopathic epiretinal membrane (iERM) is a common retinal pathology in elderly patients, thought to originate primarily from an anomalous process of posterior vitreous detachment. The standard treatment is pars plana vitrectomy (PPV) with membrane peeling. No consensus exists regarding the optimal timing of surgery, nor is it clear which patients are most likely to benefit. Given that iERM profoundly affects retinal vascular morphology and function, optical coherence tomography angiography (OCTA) has emerged as a valuable tool for identifying potential biomarkers. This systematic review aimed to synthesize the available evidence on OCTA-derived biomarkers and their correlations with visual function before and/or after surgical intervention in iERM, with a particular focus on their prognostic value for postoperative outcomes. **Methods:** A systematic search of PubMed/MEDLINE and Scopus was conducted on the 20th of May 2025 in accordance with the Preferred Reporting Items for Systematic Reviews and Meta-Analyses (PRISMA) guidelines. Eligible studies included patients with iERM undergoing vitreoretinal surgery, used OCTA for pre- and/or postoperative assessment, investigated structure–function correlations, and were designed as clinical trials, observational studies, or case series with more than 10 patients. Exclusion criteria were studies with ≤10 cases, absence of separate iERM analysis, lack of surgical intervention, or non-English language. Data extraction covered study design, demographics, surgical approach, OCTA device, follow-up, OCTA biomarkers, and structure–function outcomes. Risk of bias in observational studies was assessed using the National Institute of Health (NIH) Quality Assessment Tool for Observational Cohort and Cross-Sectional Studies. **Results:** The search yielded 1053 records, of which 71 underwent full-text review and 43 met eligibility criteria. All included studies were observational, encompassing 1958 eyes from 1953 patients. The most frequently investigated biomarkers were the foveal avascular zone (FAZ) area and related parameters, vessel density (VD), and foveal density 300 (FD-300). Additional studies evaluated average vessel length (VL), blood flow area, vessel length density (VLD), vessel tortuosity (VT), fractal dimension (FD), and perfusion capacity (PC). **Conclusions:** By consolidating current evidence, this systematic review provides a comprehensive overview of structure–function correlations in iERM and highlights the potential of OCTA-derived metrics as biomarkers of disease severity and surgical prognosis. These findings help clarify underlying mechanisms of visual decline and establish the context for further research. Nonetheless, interpretation is limited by the observational design of all included studies and by heterogeneity in OCTA methodology and nomenclature, underscoring the need for standardization to improve comparability and foster greater coherence across studies. No funding was provided for this review.

## 1. Introduction

Epiretinal membrane (ERM) is a condition characterized by the formation of a fibrocellular layer at the vitreoretinal interface [1]. It is classified as idiopathic or secondary, with the latter arising in association with coexisting or preceding ocular pathologies such as diabetic retinopathy, retinal vein occlusion, or rhegmatogenous retinal detachment. The term idiopathic indicates the absence of an identifiable underlying ocular condition, and anomalous posterior vitreous detachment has been proposed as the primary pathophysiological mechanism. Advanced age represents the most significant risk factor, as prevalence increases significantly after the age of 50 and peaks in the seventh decade of life. The clinical course of idiopathic ERM (iERM) can differ greatly between affected individuals, ranging from minimal progression and mild symptoms to rapid advancement with pronounced visual disturbance [2,3]. The membrane exerts two distinct forces on the retina, centripetal contraction and anteroposterior traction, that alter its architecture, leading to retinal thickening and wrinkling, disorganization of the retinal layers, and vascular distortion [1].

Pars plana vitrectomy (PPV) with ERM peeling is the standard treatment for iERM. However, there is no consensus regarding the optimal timing of surgery, and visual prognosis often remains uncertain despite favorable anatomical restoration, underscoring the challenges of clinical decision-making in these patients. These limitations have prompted growing interest in identifying structural predictors of functional recovery. Advances in high-resolution imaging have been central to this effort, with optical coherence tomography (OCT) transforming the diagnosis and management of iERM by allowing precise visualization of both the membrane and the underlying retinal layers. In 2017, Govetto et al. [4] proposed a four-stage OCT-based classification system for iERM using spectral-domain OCT (SD-OCT). This system is based on the presence (stage I) or absence (stage II) of the foveal pit, the presence of ectopic inner foveal layers (EIFL, stage III), and the coexistence of EIFL with disorganization of the inner retinal layers (DRIL, stage IV). Higher stages in this classification have been associated with worse visual acuity (VA) and more pronounced disruption of retinal microvasculature [4]. Mathews and coworkers proposed a three-grade classification based on alterations in foveal architecture, where grade 0 corresponds to a normal foveal depression, grade 1 to the loss of this depression, with equal retinal thickness at the fovea and surrounding macula, and grade 2 to a thickened fovea relative to the adjacent retina [5]. Although based on different criteria than Govetto’s system, it similarly captures progressive morphological changes associated with iERM severity.

Optical coherence tomography angiography (OCTA) is a non-invasive imaging modality that enables visualization of retinal blood flow without the need for intravenous contrast agents. The technique relies on motion contrast, detecting signal variations between sequential B-scans caused by erythrocyte movement within vessels while static retinal tissue remains unchanged [6]. This allows for the generation of high-resolution vascular images and enables layer-specific segmentation, providing detailed visualization of individual retinal capillary plexuses and the choriocapillaris (CC) [7]. iERM alters macular vascular architecture and dynamics, leading to shrinkage or disappearance of the foveal avascular zone (FAZ) [8], as well as changes in vascular density (VD) and flow [9]. The present study systematically synthesizes the available evidence on OCTA-derived biomarkers in iERM, with a focus on their clinical significance and their correlation with visual function, as well as their potential predictive value for postoperative outcomes. By consolidating current findings, this review seeks to aid vitreoretinal surgeons in setting more accurate expectations for postoperative VA recovery and uncover new avenues for future research. To our knowledge, this is the first systematic review to comprehensively examine OCTA biomarkers in relation to visual outcomes in iERM.

## 2. Materials and Methods

### 2.1. Literature Search Methodology

This investigation was guided by the following research question: What is the relationship between OCTA biomarkers and visual function in patients with iERM, and which of these parameters hold predictive value for visual outcomes? The systematic review was conducted in accordance with the 2020 Preferred Reporting Items for Systematic Reviews and Meta-Analyses guidelines (PRISMA). On 20 May 2025, a comprehensive literature search of PubMed/MEDLINE and Scopus was carried out using the strategies detailed in Table 1. No restrictions on publication year or study design were applied. The search results were imported into Zotero reference manager. Duplicate records were identified automatically and verified manually. Titles and abstracts were screened independently by two authors using predefined inclusion and exclusion criteria. The inclusion criteria were: (1) studies enrolling patients with iERM who underwent vitreoretinal surgery, (2) use of OCTA for preoperative and/or postoperative assessment, (3) investigation of structural–functional correlations, and (4) study designs limited to clinical trials, observational studies or case series including more than 10 patients. Studies were excluded if they (1) involved 10 or fewer cases, (2) lacked separate analysis specifically for iERM, (3) did not include surgical intervention, or (4) were published in languages other than English. Full-text articles were then reviewed in detail for eligibility by the same authors, with disagreements resolved by discussion or, when necessary, consultation with a third reviewer. From each study, the following data were extracted: author, year of publication, study design, participant demographics (age, sex), number of eyes included, type of surgical intervention, OCTA device, follow-up protocol, and OCTA biomarkers assessed. Outcome data included all available structure–function correlations at baseline and postoperative follow-up (1, 3, 6, and 12 months). When multiple time points were reported, all results were included. Missing or unclear data were retrieved from figures, tables, or supplementary materials when possible.

### 2.2. Risk of Bias Assessment Methodology

The observational studies were assessed for bias using the National Institutes of Health (NIH) Quality Assessment Tool for Observational Cohort and Cross-Sectional Studies, developed by the National Heart, Lung, and Blood Institute (NHLBI). This tool is designed to evaluate the degree to which a study’s findings can be attributed to the exposure under investigation rather than flaws in its design or conduct. It consists of 14 key questions that assess potential sources of bias or limitations across several domains: study clarity (whether the research question and study population are clearly defined), selection bias (the representativeness of the sample and the participation rate), measurement bias (how exposures and outcomes are defined, measured, and whether assessors were blinded), temporal relationships (whether exposures were measured prior to outcomes), statistical rigor (whether sample size justifications were provided and confounding factors were addressed), attrition bias (assessed through reporting on loss to follow-up). Each item was rated as Yes, No, Cannot Determine (CD), Not Reported (NR), or Not Applicable (NA). Two reviewers independently performed the quality assessments, with any discrepancies resolved by discussion. After completing the item-level evaluation, each study was assigned an overall quality rating of Good, Fair, or Poor, reflecting the reviewers’ judgment of risk of bias. The tool served as a structured framework for qualitative appraisal of methodological rigor and internal validity rather than as a numerical scoring system.

## 3. Results

**Figure 1:**
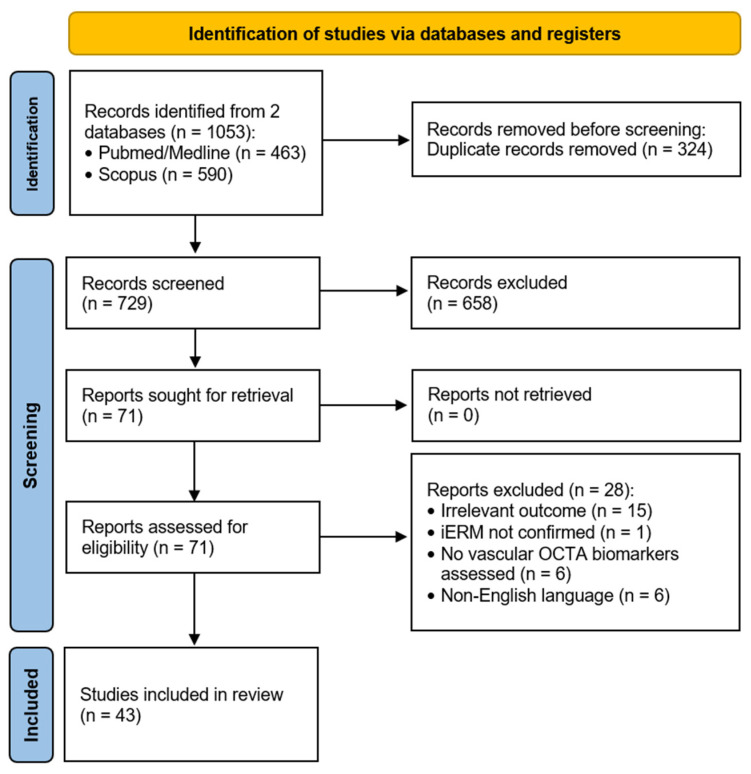
PRISMA flow diagram.

A total of 1053 records were retrieved from the two bibliographic databases, of which 324 duplicates were identified in Zotero and subsequently verified and merged manually. Title and abstract screening led to the exclusion of an additional 658 records. During full-text eligibility assessment, 28 studies were excluded due to irrelevant outcomes, lack of confirmation of the idiopathic nature of the ERM, absence of vascular OCTA biomarker assessment, or publication in a non-English language. Studies in which OCTA was applied solely for structural imaging, rather than for the evaluation of vascular biomarkers, were excluded. For example, one study analyzed inner retinal dimples using only the en face OCTA images without assessing vascular parameters [10]. Another study focused on the misalignment between the FAZ center and the foveal photoreceptor center, using OCTA to localize the FAZ but not to quantify perfusion or VD [11]. Ultimately, 43 studies were included in the systematic review. A PRISMA flow diagram chart was used to illustrate the selection process (Figure 1).

Table 2 provides an overview of the 43 studies included in this review, encompassing 1958 eyes from 1953 patients. Of these, 39 studies (involving 1796 patients) contributed to the pooled age analysis, while four were excluded due to missing mean age or standard deviation (SD) data. The calculated pooled mean age was 66.53 ± 8.21 years. Internal limiting membrane (ILM) peeling, performed in addition to iERM removal, was reported in 41 studies, whereas two either did not include ILM peeling or did not report on it explicitly. Fluid–air exchange or gas tamponade was described in nine studies, with only two specifying the use of octafluoropropane (C3F8).

### 3.1. Risk of Bias Assessment

Of the 43 included studies, all were observational in design and were assessed using the NIH Quality Assessment Tool for Observational Cohort and Cross-Sectional Studies. Nine studies were rated as good quality and three as poor. Only three studies reported sample size calculation, power analysis, or effect size estimates, indicating a general lack of statistical justification. A follow-up period of at least three months was considered sufficient to evaluate associations between OCTA parameters and visual outcomes. Two studies did not meet this threshold and were rated negatively for this item. Although some studies noted that imaging analysts were blinded to patient clinical data, none explicitly stated that outcome assessors were blinded to the exposure status of participants, leaving the potential for detection bias unaddressed. In retrospective studies where follow-up duration was part of the inclusion criteria, assessment of follow-up loss was considered NA, as complete follow-up was inherently ensured by the study design. Finally, 21 studies conducted statistical adjustment for potential confounding variables. A detailed breakdown of domain-specific responses is available in Supplementary Table S1.

### 3.2. Metrics and Terminology

A limitation in the interpretation and comparison of OCTA-derived parameters is the lack of standardized terminology [54]. The literature contains numerous terms to describe similar or even identical biomarkers. This heterogeneity extends not only to the nomenclature for vascular plexuses but also to the calculation methods for specific metrics. Table 3 summarizes the OCTA biomarkers assessed in this review, together with their widely accepted definitions.

For clarity of terminology, we designated the superficial capillary/vascular plexus/layer/complex as the “superficial capillary plexus (SCP)” and the deep equivalent as the “deep capillary plexus (DCP)”. Variability in the delineation of these plexuses contributes to methodological inconsistency. Although segmentation differences are often subtle, they may still impact quantitative outcomes. Feng et al. defined the SCP as extending from 3 μm below the ILM to 16 μm below the inner plexiform layer (IPL), and the DCP from 16 to 69 μm below the IPL [18]. Chen et al. used similar definitions, extending the DCP boundary slightly deeper to 72 μm below the IPL [26]. Zhan and colleagues applied a different approach, defining the SCP from 5 μm above the ILM to the upper third of the retinal ganglion cell complex (GCC), and the DCP from that point to 25 μm below the outer plexiform layer (OPL) [52]. Moreover, automated segmentation by the OCTA device software was commonly used, though the specific SCP and DCP boundary settings were not specified [12,20,28,48]. Given the potential influence of segmentation artifacts on the accuracy of vascular plexus measurements, consistent handling of layer segmentation is essential to ensure reliable quantification of plexus parameters. Nineteen studies explicitly reported that automated segmentation was reviewed and manually corrected when necessary [9,14,15,16,18,19,21,22,28,29,30,31,33,36,37,41,49,50,52]. An additional six studies, although not performing manual adjustments, stated that OCTA scans with segmentation failure were excluded from analysis [12,13,24,26,38,48]. The remaining studies did not specifically mention any verification of automated segmentation or exclusion criteria related to segmentation errors.

Device distribution is summarized in Table 4. The most frequently employed system was the AngioVue Imaging System (RTVue XR Avanti, Optovue Inc., Fremont, CA, USA), reported in 24 studies. Six studies used different types of OCTA devices from Carl Zeiss Meditec Inc. (Dublin, CA, USA), including both spectral-domain and swept-source models. The DRI OCT Triton (Topcon Corporation, Tokyo, Japan) was utilized in 6 studies, and NIDEK OCTA systems (NIDEK Co., Ltd., Gamagori, Japan) in 5 studies. Single studies used either the VG200 (S Vision Imaging, Luoyang, China) or the Spectralis HRA + OCT (Heidelberg Engineering, Heidelberg, Germany) with integrated OCTA capability.

Across OCTA studies, the FAZ area is among the most reproducible metrics, as most commercial devices apply comparable definitions [54]. Variability arises from differences in the retinal layers selected for measurement. Some authors quantify the FAZ area within an isolated capillary plexus, whereas others assess it using a full-thickness slab. In this review, these methodological differences are considered, and the assessment is expanded to include complementary FAZ-derived metrics that provide additional information on FAZ morphology, such as perimeter, circularity, and acircularity index (AI).

The terminology and computation of density and perfusion indices are considerably more variable. Thirty-three of the included studies evaluated such parameters, 22 of which used the AngioVue Imaging System. This device automatically calculates OCTA metrics using predefined algorithms consistent with the definitions in Table 3. Four studies instead applied ImageJ version 1.52e (an open-source platform developed by the NIH for processing and analyzing scientific images) [42] or employed other devices (DRI OCT Triton Plus, Topcon Corporation, Tokyo, Japan; VG200, S Vision Imaging, Luoyang, China), each relying on an area-based approach consistent with AngioVue [20,37,52]. Taken together, these methodological alignments provide a robust framework that enhances the reliability of VD measurements and supports cross-study comparability.

Seven studies, however, reported device-dependent differences. Four studies used the Nidek device RS 3000 Advance, which provides two distinct density metrics: (1) area-based vascular density, termed “perfusion density” by the manufacturer and defined as the proportion of the imaged area occupied by perfused vasculature (in this review, for consistency, this measure is referred to as “VD”), (2) length-based vascular density, defined as the total length of perfused vasculature per unit area (mm/mm^2^), referred to herein as “average vessel length (VL)” [13,24,33,38]. Three studies, although acquiring images with devices such as the PLEX Elite 9000 (Carl Zeiss Meditec, Dublin, CA, USA) or the DRI OCT Triton (Topcon Corporation), conducted post-processing using the ImageJ software. The reported parameters were (1) an area-based vascular density (termed “perfusion density” by the authors but reclassified here as “VD” for consistency) and (2) a length-based vascular density, defined as the ratio of total VL to total area and referred to here as “vessel length density (VLD)” [22,47,50].

## 4. Discussion

Idiopathic ERM represents a common retinal disorder, with a reported prevalence of approximately 11.8%, and its incidence increases significantly with advancing age [3]. Early investigations revealed that iERM distorts the macular capillary network, leading to increased vascular tortuosity and reduced flow velocity, changes believed to contribute to visual impairment [55]. In recent years, the introduction of OCTA has enabled non-invasive visualization and quantification of retinal microvascular alterations. Its dye-free nature has stimulated growing interest in OCTA-based biomarkers, with the aim of identifying prognostic factors for postoperative visual function and supporting clinical decision-making in iERM management.

### 4.1. FAZ-Related Biomarkers

The FAZ represents a vessel-free area located at the center of the fovea, characterized by the lack of retinal capillaries and a dense concentration of cone photoreceptors [56,57]. This structural arrangement allows unobstructed light transmission to the photoreceptors, optimizing VA by reducing optical scatter. Given its critical role in central vision, changes in FAZ morphology have been associated with impaired visual function and are increasingly recognized as potential biomarkers for assessing functional status in retinal diseases [58].

A total of 34 studies investigated FAZ-related biomarkers, with FAZ area representing the most frequently evaluated parameter. Measurements were performed either across the full retinal slab or separately within the SCP and DCP, although several studies focused exclusively on the SCP due to superior image quality and clearer vascular delineation [40]. Across the included studies, smaller FAZ area and perimeter consistently correlated with poorer best-corrected visual acuity (BCVA) both at baseline and six months postoperatively [13,14,20,21,31,33,42,43,44,45,47,53]. Further characterization of this relationship was achieved through the evaluation of interocular FAZ differences and FAZ area ratios (defined as the preoperative FAZ area of the ERM eye divided by that of the fellow eye). These metrics provided an additional layer of structural assessment and revealed that larger interocular discrepancies and lower FAZ area ratios were significantly associated with poorer BCVA [12,26,45]. Bacherini et al. reinforced Govetto’s findings by demonstrating that advanced stages of iERM are characterized by progressive FAZ constriction (FAZ area R = −0.5741; FAZ perimeter R = −0.4908), a change that was also significantly associated with reduced visual function [4,13]. Consequently, anatomical disruption, vascular remodeling, and functional impairment in iERM appear to be closely interconnected. Nevertheless, a limited number of studies reported the opposite association, suggesting that better BCVA or greater retinal sensitivity (RS) may be linked to a smaller FAZ area [20,37,53]. These findings were inconsistent and instead appeared to be confined to specific patient subgroups or postoperative time points.

The predictive value of FAZ area and perimeter for postoperative visual outcomes has been demonstrated across multiple studies, with smaller values correlating with worse BCVA postoperatively [14,49]. Recently, Zhang et al. proposed a FAZ area-based staging system derived from automated OCTA measurements on a full retinal slab extending from the ILM to 10 μm below the OPL [14]. Based on FAZ area thresholds, eyes were stratified into four stages of disease severity. This quantitative approach was developed to address the limitations of qualitative OCT classifications, which rely on subjective interpretation and are prone to intra- and inter-observer variability. The authors reported a stepwise decline in VA with advancing stage, together with a significant negative association between baseline FAZ area and BCVA at 12 months postoperatively (R = −0.30, *p* = 0.002), thereby supporting the role of FAZ area as a reliable long-term prognostic biomarker following ERM surgery.

Visual improvement has also been used to quantify surgical outcomes, most commonly expressed as the change in LogMAR BCVA (preoperative—postoperative BCVA, BCVA-d) or as Early Treatment Diabetic Retinopathy Study (ETDRS) letter score gain at six months. Studies consistently showed that eyes with smaller baseline FAZ area or perimeter, reflecting greater ERM contraction and more advanced stage, tended to exhibit greater postoperative improvement [30,41,45,49]. However, two studies by Kim et al. reported contrary findings, demonstrating instead that smaller FAZ area and perimeter were linked to less visual improvement following surgery [43,47]. This apparent inconsistency indicates that the relationship between FAZ metrics and functional recovery is likely influenced by additional factors. Previous studies have shown that ERM-induced traction can extend to compromise outer retinal integrity, particularly through disruption of the ellipsoid zone (EZ) in advanced disease [59]. When FAZ constriction occurs in the absence of EZ disruption, surgical release of the membrane alleviates mechanical stress and proportionally improves visual function. Conversely, once the EZ is damaged, visual recovery remains limited regardless of FAZ morphology. The differing patient populations across studies lend weight to this interpretation. While studies reporting greater visual improvement in eyes with smaller FAZ included relatively few stage 4 iERM cases (23.9% and 5.55%, respectively) [45,49], the majority of Kim et al.’s cohort was classified as stage 4 (67.85%), where EZ disruption is frequent [43]. This distinction may account for their contrasting findings and for the strong correlations they reported between FAZ parameters and visual outcomes (FAZ area R = 0.67, FAZ perimeter R = 0.75).

In addition to BCVA, alternative functional outcomes have been investigated in relation to FAZ parameters. Xu et al. used multifocal electroretinography (mfERG) and found that baseline FAZ area correlated with postoperative P1 amplitude in ring 1, an association that remained significant as an independent predictor in multivariate analysis [34]. Notably, three months after surgery, mfERG changes were largely confined to the central retina, with reduced response strength (lower P1 amplitude of ring 1) and faster response timing (shortened P1 implicit time). By contrast, the parameters in rings 2 through 5 did not demonstrate significant postoperative change. This selective central involvement suggests that retinal functional recovery does not occur in accordance with structural restoration or measurable improvements in VA, which may explain the persistent visual complaints reported by some patients. Hirata and colleagues evaluated the degree of aniseikonia using the New Aniseikonia Test and found significant negative correlations at 12 months postoperatively with several baseline FAZ parameters, including FAZ area, FAZ area ratio, FAZ perimeter, and FAZ perimeter ratio [39]. Of these, the FAZ area ratio emerged as an independent predictor in multivariate analysis, yielding the following regression equation: predicted postoperative degree of aniseikonia = −8.843 × preoperative FAZ area ratio + 5.755. Furthermore, they identified a superficial FAZ area ratio below 0.43 at baseline as a threshold associated with a high likelihood of developing functionally significant aniseikonia after surgery. Microperimetry was also applied to assess RS, yet no predictive correlations were identified with FAZ area or perimeter [18,38].

FAZ circularity is an index ranging from 0 to 1, where a value of 1 indicates a perfect circular shape. Among the four studies that assessed this parameter, two demonstrated that better preservation of foveal contour (greater FAZ circularity) may predict superior visual outcomes at 3 and 6 months after ERM peeling [13,47]. Evidence from other retinal conditions reinforces its relevance, as FAZ circularity has been shown to reflect terminal capillary ring integrity and may serve as a sensitive marker of microvascular damage [60]. The AI provides a complementary assessment by quantifying deviations of the FAZ outline from a perfect circle, calculated as the ratio of the measured perimeter to that of a circle with the same area. A value of 1 denotes a perfect circle, while higher values indicate increasing distortion. While most of the seven studies examining AI did not find significant correlations with VA, Zhang et al. [14] reported that higher baseline AI was associated with worse BCVA one year after surgery [16,25,27,30,31,41].

### 4.2. Vessel Density (VD)

VD is defined as the percentage of the total scanned area occupied by blood vessels [61]. Its measurement varies across studies with respect to scan size, the capillary plexus analyzed, and the specific macular subregion evaluated. Most investigations quantified VD across the entire OCTA scan area, most commonly using 3 × 3 mm^2^ or 6 × 6 mm^2^ scans. Isolated studies employed smaller (1 × 1 mm^2^) or larger (12 × 12 mm^2^) fields of view [22,38]. VD was additionally assessed within defined macular subregions, such as the fovea (1 × 1 mm^2^) or parafovea (3 × 3 mm^2^), delineated using the ETDRS grid. The perifoveal region, corresponding to the annular zone between the 3 × 3 mm^2^ and 6 × 6 mm^2^ scan boundaries, was also examined in selected studies [9,33].

Baseline cross-sectional analyses across the included literature revealed heterogeneous associations between VD and functional outcomes in iERM, with significant findings often dependent on the vascular plexus, macular subregion, and disease stage. In a prospective study, Told et al. [37] observed a strong association between foveal VD and baseline visual function, reporting that lower foveal VD correlated with poorer BCVA (R = −0.70, *p* = 0.005), but only in eyes with stage 1 or 2 ERM as classified by Govetto et al. [4]. Bacherini et al., using a 3 × 3 mm^2^ scan, stratified VD measurements by plexus and found that reduced VD in the SCP, DCP, and choroidal (CH) plexus was consistently linked to worse VA, thereby extending the relationship across all vascular layers [13]. The association between DCP impairment and visual function is not yet fully elucidated. ERM-induced distortion affects not only the inner retina but also the outer retina through tangential traction at the fovea and retrograde transneuronal degeneration [62]. As the DCP is partly responsible for supplying oxygen to the outer retina, reduced perfusion may further compromise photoreceptor function and hinder visual recovery. Furthermore, inner nuclear layer (INL) thickening has been associated with decreased VD in the DCP in the parafoveal region and poorer visual outcomes [12,63]. It is therefore hypothesized that DCP dysfunction may impair fluid clearance, leading to structural changes that disrupt synaptic transmission between photoreceptors and ganglion cells [64].

Findings concerning the SCP are notably heterogeneous, a variability likely attributable to its anatomical location. Situated within the nerve fiber and ganglion cell layers, the SCP supplies oxygen to the inner retina. These inner layers are directly affected by ERM-induced traction and particularly susceptible to mechanical stress during membrane peeling. Mao and colleagues assessed the macular vessel density ratio (MVR), defined as the ratio of foveal to parafoveal VD, proposing it as a potentially more sensitive metric for quantifying alterations in capillary architecture [31]. Higher MVR values were indicative of greater structural distortion and, consequently, more severe visual impairment. A positive correlation between MVR in the SCP and LogMAR BCVA was reported. These results align with findings from Feng and coworkers, who noted that higher foveal SCP VD was associated with worse RS at baseline [18]. Such an observation is likely explained by greater centripetal displacement of SCP vasculature into the foveal region under stronger iERM traction, thereby increasing measured VD.

At 3 months postoperatively, studies consistently demonstrated that reduced SCP VD is associated with poorer visual function, as measured by both BCVA and macular sensitivity (MS). The postoperative reductions in SCP VD may reflect microvascular disruption or structural injury, serving as an indirect marker of surgical impact on retinal integrity. Shen et al. reported that lower SCP VD correlated with worse BCVA and reduced mean RS [16]. Xu et al. observed positive correlations between SCP VD and RS across the entire 6 × 6 mm^2^ scan area, as well as within the parafoveal and perifoveal regions, though these associations were confined to patients with grade 2 iERM according to Mathew’s OCT-based classification [9]. This may reflect a greater risk of microvascular damage during membrane peeling in more advanced disease stages, where retinal adhesion is more pronounced, potentially leading to direct impairment of RS. D’Aloisio and colleagues extended their analysis beyond the central macula to include the midperiphery, defined as three 3-mm-diameter sectors (temporal, superior, and inferior) surrounding the macular region. Within the macula (5-mm-diameter annulus), they found a very strong positive correlation (r = 0.800) between SCP VD and MS at both 10° and 2°. In contrast, in the midperiphery, significant correlations with MS at 10° were observed only for DCP VD [22]. In line with their earlier findings, Bacherini et al. reported that lower CH VD remained significantly associated with worse BCVA at 3 months after surgery [13].

Correlations at 6 months may be more reliable, as retinal structure and function continue to recover gradually over time, allowing a more stable assessment of postoperative outcomes [65]. Kim and colleagues employed interocular comparisons to account for confounding variables and demonstrated that greater reductions in parafoveal VD in both the SCP and DCP were strongly associated with poorer BCVA (R = 0.657 and R = 0.633, respectively) [12]. In the same analysis, they also identified a correlation between reduced parafoveal SCP VD and thinning of the parafoveal GCC after surgery, suggesting neuronal damage resulting either from ERM-induced mechanical distortion with secondary vascular compromise or from the surgical intervention itself. Regardless of the mechanism, such alterations contribute to subsequent visual decline. Likewise, in a prospective study, Isik-Ericek and colleagues found that reduced parafoveal DCP VD at 6 months was associated with poorer BCVA, indicating a more pronounced vulnerability of the DCP to ERM-related damage and its influence on visual function [15].

The predictive value of VD parameters for postoperative visual function was evaluated in seventeen studies, of which nine reported significant predictive correlations. Feng et al. and Li et al. demonstrated that higher baseline foveal DCP VD predicts better BCVA at three months, while Xu and colleagues demonstrated the same relationship for parafoveal VD [18,34,49]. In addition, Feng and coworkers reported that increased baseline foveal DCP VD independently predicts improved foveal and parafoveal sensitivity at the same postoperative interval, a finding confirmed in an age and gender–adjusted multivariate linear regression model [18]. These findings emphasize the critical role of the DCP in maintaining visual function. Given that the DCP contributes to the vascular supply of the outer retina, disruption of this network, accompanied by edema and ischemia within the pathological process, may intensify outer retinal damage and ultimately lead to irreversible photoreceptor dysfunction.

Furthermore, Li et al. also investigated the degree of postoperative visual improvement, defined as the difference between baseline and postoperative BCVA, and its relationship with VD parameters. Interestingly, they found that higher foveal SCP VD (*p* < 0.0001) and lower foveal DCP VD were associated with greater visual gains [49]. As previously discussed, more advanced iERM may cause pronounced central displacement of the capillary network, particularly within the SCP, which lies closest to the inner retinal surface, thereby increasing measured foveal VD. Consequently, these findings may suggest that patients with more severe iERM, who present with higher apparent foveal VD in the SCP due to vascular displacement, may experience greater visual improvement following surgery. This observation was further supported by Mao et al., who likewise reported that higher foveal SCP VD was associated with greater visual improvement, albeit at six months postoperatively [41].

ERM appears to exert effects beyond the SCP and DCP, propagating downstream to influence the CH microvasculature [66]. Such alterations have been observed both prior to surgery and throughout postoperative follow-up. Bacherini et al. [13] evaluated VD in both the entire CH and specifically within the CC. Of these, only CHVD demonstrated predictive value for visual function at 3 months, as lower baseline values were associated with poorer BCVA [13]. In a prospective study, Rommel and colleagues extended this approach by stratifying the CH into its distinct layers, namely CC, Sattler’s layer (SL), and Haller’s layer (HL), which lie progressively farther from the retina and contain vessels of increasing caliber [50]. They reported that only the VD in the SL predicted BCVA at 3 months, with higher VD correlating with better outcomes. At the same time, postoperative thickening of the CH at 3 months was largely attributable to the CC, where VD increased (*p* = 0.003), while VD in the SL declined (*p* = 0.014). Vitrectomy is known to induce several physiological changes in intraocular oxygenation and VEGF levels, which may influence CH hemodynamics. The postoperative pattern observed here may therefore represent adaptive vascular remodeling, with blood flow redistributed from larger-caliber vessels toward the finer capillary network of the CC. In this context, the predictive role of SL may lie in its ability to indicate the reserve capacity of CH perfusion that can be recruited into the CC following surgery. By contrast, Kim et al., who focused exclusively on the CC, reported conflicting findings: although higher baseline CCVD was associated with greater visual improvement at 6 months, it simultaneously predicted poorer anatomical and functional outcomes [47]. Interpretation of these results, however, is limited by the retrospective design, the small cohort size, and the inherent challenges of segmenting the CC, which consists of very small vessels and is more difficult to delineate compared with other CH layers [67]. Other studies assessing the CC did not demonstrate correlations with visual function, although predictive analyses were lacking [22,26]. Nevertheless, these observations may point to a potential role of preserved CH perfusion in maintaining retinal function under macular stress and supporting visual recovery.

Interestingly, a recent prospective study by Mastrogiuseppe and colleagues extended the evaluation of predictive vascular biomarkers beyond the macula to the optic nerve head (ONH) [46]. They analyzed VD parameters of the radial peripapillary capillaries (RPCs), including whole RPC VD (wRPC; measured across the entire 4.5 × 4.5 mm^2^ image centered on the optic disc), inside-disc RPC VD, and peripapillary RPC VD (measured in a 750 µm wide annulus surrounding the disc). In multivariate linear regression analysis, higher baseline wRPC VD emerged as an independent predictor of postoperative visual improvement at 12 months. This observation points to a potential role of ONH microvasculature in visual recovery after ERM surgery. It may reflect retinal nerve fiber layer (RNFL) integrity or indicate reversible axonal damage in eyes with a healthier optic nerve.

### 4.3. Foveal Density-300 (FD-300)

FD-300 is an OCTA-derived biomarker that quantifies VD within a 300-µm-wide annulus surrounding the FAZ. It is regarded as a more reliable indicator of foveal microvascular status than conventional foveal VD, as it is not influenced by FAZ size or shape [68]. This biomarker was assessed in eight studies, all of which employed the same OCTA device (Optovue RTVue XR Avanti, Optovue Inc., Fremont, CA, USA). Zhang and associates reported that lower FD-300 at baseline was associated with poorer baseline BCVA and also predicted worse BCVA at 12 months after surgery in stage 2 and stage 3 eyes, as classified by FAZ area (stage 2: 0.16 mm^2^ > FAZ ≥ 0.08 mm^2^; stage 3: 0.08 mm^2^ > FAZ ≥ 0.04 mm^2^) [14]. Supporting this, Li et al. reported a similar negative association between FD-300 and LogMAR BCVA at 12 months in a multivariate linear regression model (t = −2.807, *p* = 0.011) [25]. These data indicate that compromised parafoveal microvasculature may exert a long-term influence on visual recovery, possibly due to the slow postoperative restoration of capillary networks around the FAZ. However, the remaining studies assessing FD-300 reported no significant cross-sectional or predictive correlations with visual outcomes [27,28,30,31,41,49].

### 4.4. Average Vessel Length (VL)

The average VL represents the cumulative length of perfused vessels within the scanned area, expressed as millimeters per square millimeter (mm/mm^2^; mm^−1^). This parameter was evaluated in four studies, all conducted with the NIDEK OCTA devices and reported both cross-sectional and predictive correlations. While minor discrepancies were noted across analyses, the consistent finding was that shorter VL correlated with poorer visual function.

Henry et al. assessed the average VL across the entire 3 × 3 mm^2^ macular scan and reported that reduced DCP VL at baseline correlated with worse BCVA [33]. They further applied Pearson correlation to explore predictive associations, finding significant negative correlations between baseline macular VL in both the SCP and DCP and BCVA at six months. In bivariate analysis, SCP VL remained an independent predictor (β = −0.024 ± 0.012, *p* = 0.042). However, neither association retained significance after multivariate adjustment. Bacherini and colleagues corroborated the baseline observation by demonstrating that lower DCP VL was negatively correlated with BCVA and further extended this finding to the CH plexus, where the association persisted at three months postoperatively [13]. In addition, they observed a gradual increase in VL and VD that continued up to six months after surgery.

Osada and colleagues focused their analysis on foveal average VL within a central 1 mm^2^ area and found that, at six months postoperatively, DCP VL positively correlated with RS [38]. Although preoperative values did not predict postoperative RS, higher DCP VL at one month was associated with better RS at six months, and this relationship remained significant after adjustment for potential confounders. By contrast, Nicolai et al., using a larger 4.5 × 4.5 mm^2^ scan area, reported different associations at six months. In their cohort, BCVA showed a negative correlation with VL in the SCP and CC [24]. To further explore these associations, they stratified patients based on postoperative RS into “Improved” and “Worsened/Unchanged” groups. They found a significantly greater postoperative increase in VL across the SCP, DCP, and CC within both the foveal and parafoveal regions in the “Improved” RS group in comparison to those with stable or worsened RS.

### 4.5. Blood Flow Area

Blood flow is defined as the area or intensity of flow signal within a predefined region of interest [69]. Compared with VD or average VL, this parameter demonstrates a broader dynamic range for detecting physiological alterations in perfusion and may capture subclinical flow disturbances that precede overt capillary loss [70]. The Optovue system, equipped with the split-spectrum amplitude decorrelation algorithm (SSADA), enables automated blood flow quantification, a feature reported in six studies. The most commonly applied region of interest was a 1 mm-radius circular area centered at the fovea, corresponding to an area of approximately 3.14 mm^2^. An investigation of flow area in the SCP and DCP showed that reduced values in the DCP were significantly associated with poorer BCVA at 6 months after surgery (R = −0.52, *p* = 0.01) [15]. Complementing these findings, a large prospective study of 102 patients by Wang et al. focused on the CH and similarly demonstrated that diminished CC flow correlated with reduced visual function at 1, 3, and 6 months postoperatively [36]. Moreover, greater postoperative improvements in CC perfusion were accompanied by better BCVA at the same time points. Although no structural alterations of the CH were identified, affected eyes exhibited lower baseline flow compared with controls, with a measurable postoperative increase likely reflecting improved oxygenation. These results provide compelling evidence that ERM-induced mechanical stress is not confined to the inner retinal surface but extends to deeper layers, including the CC, and that such vascular disturbances are functionally relevant through their impact on photoreceptor integrity. The remaining studies did not demonstrate significant correlations with visual outcomes, which in some instances may reflect differences in the selected region of interest and the way flow was quantified [23,26,35,46].

### 4.6. Other Indices

Vessel length density (VLD). D’Aloisio and colleagues additionally assessed VLD as an alternative OCTA-derived metric to VD [22]. While VD measures the proportion of area occupied by vessels, VLD captures the total length of perfused vasculature by reducing each vessel to a single-pixel line. This method assigns equal weight to large and small vessels, potentially increasing sensitivity to capillary-level changes [71]. Significant correlations were observed between macular SCP VLD, assessed within a 5 mm area around the fovea, and MS at both 10° and 2°, while in the midperiphery, covering the temporal, superior, and inferior sectors adjacent to the macula, MS was associated with DCP VLD.

Vessel tortuosity (VT). In eyes with ERM, tangential and vertical macular traction reshapes the retinal vasculature, resulting in vessel displacement and increased tortuosity [72]. Surgical release of this traction through vitrectomy with ERM peeling restores the vitreoretinal interface, facilitating vascular remodeling and subsequent visual improvement [19]. Various approaches have been employed to quantify the degree of vascular distortion on OCTA en face images. VT was evaluated in two studies, both using the definition provided in Table 3. These analyses focused on the SCP within a 6 × 6 mm^2^ scan area and measurements were obtained from skeletonized OCTA images processed in ImageJ. Yanık et al. observed that one month after surgery, VT positively correlated with BCVA, although predictive value was not assessed [27]. When comparing ERM removal alone with combined ERM and ILM peeling, no significant differences in postoperative VT were detected, which may indicate that ERM peeling itself is adequate to release microvascular traction. Shen and colleagues, however, subdivided the macular area into four quadrants and, using multivariate linear regression, showed that reductions in VT within the temporal, superior, and inferior quadrants over a 3-month postoperative period correlated with improvements in MS within the central 20° field centered on the fovea [16].

Miyazawa et al. analyzed VT at the level of individual retinal vessels [19]. Using ImageJ for image processing, they divided a 6 × 6 mm^2^ scan into four quadrants, and in each quadrant a large retinal vessel was randomly selected. Vessel distortion was quantified by comparing the actual VL between two branch points with the direct straight-line distance between them (BD), yielding a ratio that reflects vessel curvature (VL/BD). After ERM removal, VL/BD values decreased, indicating straighter vessel morphology following surgery, and these changes also showed significant associations with visual function. In particular, VL/BD in the superior and inferior quadrants correlated positively with postoperative BCVA at 1, 3, and 6 months, while changes in VL in the nasal quadrant were linked to BCVA improvements at 3 and 6 months. VL/BD thus emerges as a promising marker of vascular remodeling with potential utility in predicting postoperative visual recovery.

Although not directly quantifying VT, Li and colleagues qualitatively graded it on OCTA en face images using a five-level scoring system [49]. A score of “0” indicated clearly visible vessels without tortuosity, while higher scores reflected progressively greater small-vessel tortuosity, blurring, and reduction in recognizable retinal circulation, with “4” representing severe tortuosity and complete loss of vascular detail. They observed that VT increased in parallel to advancing iERM stage as defined by the Govetto classification, whereas postoperative tortuosity improved across all stages and showed the greatest recovery in stage 4 eyes. Furthermore, tortuosity scores correlated with visual outcomes. Eyes showing less tortuosity achieved better postoperative VA, although the relative degree of improvement was smaller compared with more advanced stages.

Fractal dimension (FD). Kim and Park investigated whether parafoveal capillary architecture could predict visual outcomes after ERM surgery, analyzing fractal dimension (FD) and lacunarity in the DCP, and foveal branch point (FBP) length in the SCP. The FBP length was defined as the mean distance from the foveal center to the nearest vessel branching points within the parafoveal ring [29]. Their results highlighted the profound impact of ERM on the DCP vasculature, with reduced vascular complexity (lower FD) and larger flow voids (higher lacunarity) both associated with worse BCVA and greater metamorphopsia. Importantly, FD also demonstrated predictive value, as baseline FD negatively correlated with BCVA at 1 week, 1 month, and 4 months postoperatively. This association persisted also at 10 months in the pseudophakic subgroup, remaining significant in multivariate analysis. Beyond this, in conditions such as diabetic retinopathy and retinal vein occlusion, FD reductions have been linked to vascular rarefaction and loss of branching complexity, while in diabetes specifically the measure has been suggested as an early marker of microvascular alteration [73,74,75]. Consequently, the reductions in FD resemble those observed in iERM, possibly reflecting a common pathway of vascular compromise across different retinal pathologies.

Perfusion capacity (PC). Recognizing that traditional OCTA metrics such as FAZ parameters, VD, FD, and VT are especially prone to confounding by structural alterations like macular edema or vascular congestion, Zhan and colleagues proposed a novel biomarker termed PC [52]. This index integrates VD with perfusion area (PA). The latter represents the absolute area of active blood flow in square millimeters. The index is calculated as the ratio of PA to the product of VD and the scan area. In their study, PC was measured within the inner 3 mm and 6 mm ETDRS circles, with lower values observed in the SCP of eyes exhibiting more severe vascular distortion. At three months postoperatively, BCVA showed a significant correlation with PC in the 6 × 6 mm^2^ SCP region (R = −0.42, *p* = 0.021). Moreover, higher baseline SCP PC values predicted greater postoperative improvements in RS, and multivariate analysis confirmed that preoperative SCP PC in the 3 × 3 mm^2^ region was independently associated with RS outcomes at follow-up. By adjusting perfusion for VD, PC may better capture dynamic changes in retinal perfusion in the context of structural distortion.

### 4.7. Limitations and Future Perspectives

OCTA has become an increasingly valuable imaging modality in both research and clinical practice. However, interpretation and comparability of its quantitative outcomes remain hindered by substantial methodological variability. The main sources of inconsistency arise from differences in segmentation algorithms and vascular boundary definitions across imaging devices and analysis software. This study highlights the need to prioritize methodological standardization in OCTA acquisition and analysis, not only in eyes affected by epiretinal membrane but across all retinal pathologies. Uniform definitions of vascular plexus boundaries, harmonized segmentation protocols, and consistent manual verification of automated outputs are essential to reduce inter-study variability and enhance the reliability of quantitative measurements. Consensus is also needed regarding optimal scan dimensions and metrics for specific biomarkers (for example, the use of 6 × 6 mm^2^ versus 12 × 12 mm^2^ scans for VD assessment or clarification of whether FAZ quantification should be restricted to the SCP or extended to the full retinal slab). Equally important is the adoption of standardized terminology, as identical terms are often used across studies to describe parameters that capture distinct vascular characteristics. The development of consensus-based imaging guidelines would substantially improve reproducibility and accelerate the clinical translation of OCTA-derived biomarkers.

This review has several limitations. First, design-related limitations may have introduced bias across the included studies. Most comprised small observational cohorts with predominantly retrospective designs and relatively short follow-up periods. The overall risk of bias was moderate in most cases, primarily due to the absence of essential methodological elements such as sample size justification and power analysis. Furthermore, only about half of the studies implemented statistical adjustments for potential confounding variables, which may have affected the robustness of the reported associations. Substantial heterogeneity in study populations and participant characteristics, including age and disease duration, as well as in OCTA devices and follow-up intervals, may have served as sources of bias and account for the observed variability in reported outcomes. Second, the review process is inherently subject to certain limitations. Restricting inclusion to studies published in English may have introduced language bias and constrained the generalizability of the findings. Although a comprehensive search strategy was implemented across two major databases, the possibility remains that relevant unpublished or non-indexed literature was not identified, potentially contributing to publication bias. Finally, due to variability in study designs and reported outcomes, a meta-analysis was not feasible. As a result, findings were synthesized narratively. These limitations should be considered when interpreting the conclusions of this review.

## 5. Conclusions

Across the reviewed literature, multiple OCTA-derived biomarkers exhibited consistent associations with visual function. FAZ area and perimeter correlated with concurrent BCVA and also demonstrated prognostic relevance, with smaller baseline values associated with worse preoperative and postoperative BCVA. Moreover, smaller FAZ dimensions appeared to predict greater postoperative visual improvement, although this relationship may be modulated by the integrity of the outer retinal layers. VD within the DCP and the CH was linked to superior postoperative BCVA and enhanced foveal and parafoveal RS, highlighting the importance of deeper microvascular integrity in visual recovery. FD-300 emerged as a promising novel biomarker with potential predictive value for postoperative outcomes. Other parameters, including average VL, blood flow area, and VT, showed primarily cross-sectional correlations with visual function, and their prognostic significance warrants validation in larger longitudinal studies. Progress in this field, however, remains limited by heterogeneity in imaging devices, inconsistent parameter definitions, and variability in analyzed retinal regions. Nonetheless, the variability which is generated by the differences between vitreoretinal surgeons is very difficult to overcome when analyzing the results in the postoperative setting.

This review has synthesized the most extensively studied OCTA metrics in iERM and highlighted novel candidate biomarkers whose clinical relevance remains to be validated. Emphasis should be placed on predictive biomarkers, as no consensus currently exists on the optimal timing or indications for surgery. Reliable OCTA-derived parameters could ultimately guide surgical decision-making, facilitate the identification of patients most likely to benefit from intervention, and provide prognostic value regarding long-term visual outcomes.

## Figures and Tables

**Table 1:** Search strategies for each database.

Database	Strategy	Results
Pubmed	(“membrane”[all fields] AND “epimacular”[all fields]) OR (“membrane”[all fields] AND “epiretinal”[all fields]) OR (“cellophane”[all fields] AND “maculopathy”[all fields]) OR (“macular”[all fields] AND “pucker”[all fields]) AND (“peeling”[all fields] OR “surgery”[all fields]) AND (“OCTA”[all fields] OR “angiography”[all fields])	463
Scopus	((membrane AND epimacular) OR (membrane AND epiretinal) OR (cellophane AND maculopathy) OR (macular AND pucker) AND (peeling OR surgery) AND (octa OR angiography))	590

**Table 2:** Summary of research studies on structure–function correlations in iERM.

Study	Study Design	No. of iERM Eyes Included	Control Group	Type of Surgery	Follow-Up OCTA Regimen	Analyzed OCTA Biomarkers	OCTA Biomarkers Correlated with Visual Function
Kim et al., 2018 [12]	Retrospective	43	Fellow healthy eye	PPV with ERM and ILM peeling + Phacoemulsification and foldable IOL implantation in phakic eyes	6 months	**FAZ area in the SCP and DCP**, **Parafoveal VD in the SCP and DCP**	**6 months**: Greater decreases in FAZ area and parafoveal VD both in the SCP and DCP compared to the fellow eye correlated with worse BCVA
Bacherini et al., 2021 [13]	Prospective	23	None	PPV 25G with ERM and ILM peeling + Gas tamponade + Phacoemulsification and IOL implantation	Baseline, 1, 3, 6 months	**FAZ area**, **FAZ perimeter**, **FAZ circularity**, **VD and PD in the SCP**, **DCP**, CC and **CH**	**Baseline**: Lower SCPPD, DCPPD, DCPVD, CHPD, CHVD, FAZ area and FAZ perimeter correlated with lower BCVA **1 month**: BCVA negatively correlated with FAZ area and FAZ perimeter **3 months**: BCVA negatively correlated with CHPD, CHVD, FAZ circularity **6 months**: BCVA correlated with SCPVD (inverse finding—higher SCPVD associated with worse BCVA) **Predictive**: Baseline FAZ circularity and CHPD negatively correlated with BCVA at 3 months
Zhang et al., 2024 [14]	Retrospective	162 (105 had surgery and the 12-month follow-up)	Fellow healthy eye	PPV 23G with ERM and ILM peeling + Phacoemulsification and IOL implantation in phakic eyes	Baseline, 1, 3, 6, 12 months	**FAZ area**, **FAZ perimeter**, **FAZ AI**, **FD-300** on the retina slab	**Baseline**: FAZ AI positively correlated with BCVA across all stages; FAZ area, FAZ perimeter and FD-300 negatively correlated with BCVA generally, across all stages, and at stage 1 iERM (FAZ-based classification) **Predictive**: Baseline FAZ area (over all stages) and FD-300 (overall stages and stages 2 and 3) negatively correlated with final BCVA
Isik-Ericek et al., 2021 [15]	Prospective	24	Age- and sex-matched group	PPV 23G with ERM ± ILM peeling + Fluid/air exchange ± Gas tamponade + Phacoemulsification and IOL implantation in case of advanced lens opacities	Baseline, 1, 3, 6 months	FAZ area, **Parafoveal VD in the** SCP and **DCP**, **flow area** in the SCP and **DCP**	**6 months**: Parafoveal VD and flow area, both in DCP negatively correlated with BCVA
Shen et al., 2023 [16]	Retrospective	41	Age-matched group	PPV 23G with ERM and ILM peeling + Phacoemulsification and IOL implantation in phakic eyes over 55 years of age	Baseline, 1, 3 months	FAZ area, FAZ perimeter, FAZ AI, **VD in the SCP** and DCP, **VT**	**3 months**: SCP VD negatively correlated with BCVA and positively correlated with MS **Predictive**: Reduction in VT in the temporal, superior, and inferior quadrants over a 3-month period correlated with improvement of retinal MS in multivariate linear regression
Okawa et al., 2019 [17]	Retrospective	49 (20 had surgery)	Age-matched group	PPV with ERM and ILM peeling + Phacoemulsification and IOL implantation in phakic eyes	Baseline & final (mean 147 d); follow-up <6 vs. ≥6 months	FAZ area in the SCP	None identified
Feng et al., 2021 [18]	Retrospective observational	25	None	PPV 23G with ERM and ILM peeling + Fluid/air exchange + Phacoemulsification and IOL implantation in phakic eyes	Baseline, 3 months	FAZ area, **VD in the SCP and DCP in the fovea and parafovea**	**Baseline**: VD of foveal and parafoveal SCP negatively correlated with foveal sensitivity **Predictive**: Higher baseline foveal VD of the DCP predicts better BCVA and higher foveal and parafoveal sensitivity at 3 months postoperatively (confirmed by a multiple linear regression model)
Miyazawa et al., 2022 [19]	Prospective	22	None	PPV 25G with ERM and ILM peeling + Phacoemulsification and IOL implantation in phakic eyes	Baseline, 1, 3, 6 months	FAZ area in the SCP and DCP, **Distortion of vessels (VL/BD)**	**1, 3, 6 months**: VL/BD in the superior and inferior quadrants was positively correlated with postoperative BCVA **Predictive**: Change in VL in the nasal quadrant was positively associated with change in BCVA at 3 and 6 months
Yuce et al., 2021 [20]	Retrospective	22	Fellow healthy eye	PPV 25G with ERM and ILM peeling ± Phacoemulsification and IOL implantation	Baseline, 6 months	**FAZ area in SCP and DCP**, **VD in the** SCP and **DCP in the fovea and parafovea**	**Baseline**: BCVA negatively correlates with FAZ area in SCP and DCP **6 months**: BCVA positively correlates with FAZ area in SCP and DCP **Baseline and 6 months**: VD DCP in the fovea and parafovea positively correlated with BCVA
Bae and Ryoo, 2022 [21]	Retrospective	43	Fellow healthy eye	PPV 25G with ERM and ILM peeling + Phacoemulsification and IOL implantation in phakic eyes	3 months before, baseline, 1, 3, 6, 12 months	**FAZ area in the SCP**	**Baseline**: FAZ area was negatively correlated with BCVA
D’Aloisio et al., 2021 [22]	Observational	23	None	PPV 25G with ERM and ILM peeling + Phacoemulsification and IOL implantation in phakic eyes	Baseline, 1, 3 months	**PD in the SCP, DCP** and CC, **VLD in the SCP and DCP**	**3 months**: macular SCP PD positively correlated with MS at 10° and 2°, macular DCP PD with MS at 2°, and peripheral DCP PD with MS at 10°. Similarly, macular SCP VLD positively correlated with MS at both 10° and 2°, and peripheral DCP VLD with MS at 10° and 2°
Li et al., 2019 [23]	Prospective	24	None	PPV with ERM and ILM peeling + Air or Gas tamponade	Baseline, 3 months	VD of the CCP, flow area in the SCP, DCP and CCP	None identified
Nicolai et al., 2024 [24]	Prospective, observational	29	Fellow healthy eye	PPV 27G with ERM and ILM peeling + Phacoemulsification and IOL implantation in phakic eyes	Baseline, 6 months	FAZ area, **VPD in the SCP, DCP and CC**	**6 months**: BCVA negatively correlated with postoperative VPD in the SCP and CC plexus **Predictive**: Patients with improved RS postoperatively showed significantly greater increases in VPD in the SCP, DCP, and CC within both the foveal and parafoveal regions
Li et al., 2023 [25]	Retrospective	74 (36 with OCTA at 12-month follow-up)	None	PPV 23G with ERM and ILM peeling + Phacoemulsification and IOL implantation in phakic eyes	12 months	FAZ area, FAZ perimeter, FAZ AI, **FD-300**	**12 months**: FD-300 negatively correlated with BCVA (in a multiple linear regression analysis)
Chen et al., 2019 [26]	Observational	33	Fellow healthy eye	PPV with ERM peeling	Baseline, 6 months	**FAZ area in the SCP**, VD in the fovea and parafovea in the SCP, DCP, OCP and CCP, flow area in the OCP and CCP	**6 months**: FAZ area negatively correlated with BCVA and larger interocular differences in FAZ area correlated with worse BCVA
Xu et al., 2021 [9]	Retrospective	53 (35 had surgery)	22 eyes with mild cataract only	PPV 23G with ERM and ILM peeling + Phacoemulsification and IOL implantation in phakic eyes age over 50	Baseline, 3 months	**VD in the SCP** and DCP in the fovea, parafovea, perifovea and whole VD (6 × 6 mm^2^ area)	**3 months**: whole RS positively correlated with whole VD, parafoveal RS with parafoveal VD, and perifoveal RS with perifoveal VD, all within the SCP, only in eyes classified as grade 2 iERM according to the Mathews OCT classification
Yanık et al., 2023 [27]	Retrospective	25	None	PPV 25G with ERM ± ILM peeling (15 had ERM + ILM peeling)	Baseline, 1 month	FAZ area, FAZ perimeter, FAZ AI, FD-300, **RVTI** in the SCP	**1 month**: BCVA correlated with RVTI
Caretti et al., 2025 [28]	Retrospective	39	None	PPV 27G with ERM and ILM peeling + Phacoemulsification and IOL implantation in phakic eyes (all patients)	Baseline, 1, 6 months	FAZ area, FAZ perimeter, FD-300 on the retina slab, VD in the SCP and DCP in the fovea and parafovea	None identified
Kim and Park, 2021 [29]	Retrospective	71	None	PPV 25G with ERM and ILM peeling + Phacoemulsification and IOL implantation in eyes with visually significant cataract	Baseline, 1 week, 1, 3, 6 months	**FD and lacunarity in the parafoveal DCP**, **FBP** length and **difference in the SCP**	**Baseline**: BCVA negatively correlated with FD and positively with lacunarity in the DCP and FBP difference in the SCP; Metamorphopsia negatively correlated with FD and positively with lacunarity in the DCP **Predictive**: Baseline FD in the DCP negatively correlated with BCVA at 1 week and 1 and 4 months after surgery (significant correlation at 10 months in the pseudophakic group that remained significant in multivariate analysis)
Ersoz et al., 2021 [30]	Retrospective	28 (included patients with intact EZ only)	None	PPV 23G with ERM and ILM peeling	Baseline, 6 months	**FAZ area**, **FAZ perimeter**, FAZ AI, FD-300 on the retina slab	**Predictive**: Postoperative letter score gain correlated negatively with baseline FAZ area and FAZ perimeter (FAZ perimeter confirmed by multivariable linear regression analysis)
Mao et al., 2021 [31]	Retrospective	100 (62 had surgery)	Fellow healthy eye	PPV with ERM and ILM peeling + Phacoemulsification and IOL implantation in phakic eyes over 50 age	Baseline, 3 months	**FAZ area**, **FAZ perimeter**, FAZ AI, FD-300, SCP VD and **DCP VD in the fovea** and parafovea, **MVR (FVD/PRVD) in the SCP** and DCP	**Baseline**: BCVA negatively correlated with FAZ area and FAZ perimeter and positively correlated with the MVR in the SCP **Predictive**: Postoperative BCVA positively correlated with baseline foveal VD in the DCP
Chatzistergiou et al., 2021 [32]	Retrospective	54	None	PPV with ERM peeling	Baseline, 3 months	VD in the SCP and DCP of the whole image (6 × 6 mm^2^ area) and of the fovea	None identified
Henry et al., 2024 [33]	Retrospective observational	47	Fellow healthy eye	PPV 25G with ERM and ILM peeling + Phacoemulsification and IOL implantation in phakic eyes over 60 age or younger in case of cataract	Baseline, 1 week, 1, 6 months	**FAZ area in the SCP**, **VD in the SCP and DCP in the whole macular region** (3 × 3 mm^2^), fovea, parafovea, and perifovea	**Baseline**: BCVA negatively correlated with FAZ area and macular VD in the DCP **Predictive**: BCVA at 6 months was negatively correlated with baseline macular VD in both the DCP and SCP; neither association remained significant in multivariate regression analysis
Xu et al., 2024 [34]	Retrospective	30	None	PPV 25G with ERM and ILM peeling + Phacoemulsification and IOL implantation in phakic eyes	Baseline, 1, 3 months	**FAZ area**, **VD in the** SCP and **DCP** in the fovea and parafovea	**Predictive**: Baseline parafoveal VD in DCP negatively correlated with postoperative BCVA and baseline FAZ area positively correlated with postoperative P1 amplitude (ring 1); both correlations persisted as independent predictors in the multivariate linear regression analysis
Hondur and Aribas, 2024 [35]	Retrospective	33	Fellow healthy eye	PPV 25G with ERM and ILM peeling + Fluid/air exchange (air/ gas SF6 exchange performed in 1 eye with retinal tear)	Baseline, 6 months	CC flow density	None identified
Wang et al., 2023 [36]	Prospective	102	Fellow healthy eye	PPV 25G with ERM and ILM peeling + Phacoemulsification and IOL implantation in phakic eyes	Baseline, 1 week, 1, 3, 6, 12 months	**Choroidal capillary perfusion**	**1, 3, 6, 12 months**: CC perfusion and ΔCC perfusion negatively correlated with BCVA
Told et al., 2020 [37]	Prospective	32	Fellow healthy eye	PPV 23G with ERM and ILM peeling + Phacoemulsification and IOL implantation in phakic eyes with cataracts	Baseline, 1 day, 1 week, 1, 3 months	**FAZ area in the SCP** and DCP, **VD in the fovea** and parafovea	**Baseline**: BCVA was negatively correlated with foveal VD in stage 1-2 iERM **Week 1**: BCVA was positively correlated with FAZ area in SCP in stage 3–4 iERM
Osada et al., 2020 [38]	Retrospective	25	Fellow healthy eye	PPV 27G with ERM and ILM peeling + Phacoemulsification and IOL implantation in phakic eyes	Baseline, 1, 3, 6, 12 months	FAZ area in the SCP, **VD in the fovea in the** SCP and **DCP**	**6 months**: BCVA and RS positively correlated with foveal VD in the DCP **Predictive**: BCVA and RS at 6 months were positively correlated with foveal DCP VD at 1 and 3 months; higher foveal DCP VD at 1 month was an independent predictor of better RS at 6 months in the multivariate analysis
Hirata et al., 2019 [39]	Prospective	30	Fellow healthy eye	PPV 25G with ERM and ILM peeling + Phacoemulsification and IOL implantation in phakic eyes	Baseline, 1, 3, 6, 12 months	**FAZ area**, **FAZ perimeter**, FAZ circularity in the SCP, **FAZ area ratio and FAZ perimeter ratio (interocular ratios)**	**12 months**: Aniseikonia negatively correlated with FAZa, FAZa ratio, FAZp, and FAZp ratio **Predictive**: Aniseikonia at 12 months negatively correlated with baseline FAZa, FAZa ratio, FAZp, and FAZp ratio; baseline FAZa ratio was identified as an independent predictor in multivariate analysis
Honzawa et al., 2023 [40]	Retrospective	37	Fellow healthy eye and 26 healthy eyes	PPV with ERM and ILM peeling + Phacoemulsification and IOL implantation in phakic eyes (33)	6 months	**FAZ area in the SCP**	**Predictive**: Measured FAZ area changes were negatively correlated with baseline BCVA
Mao et al., 2020 [41]	Prospective	35	Fellow healthy eye	PPV 23G with ERM and ILM peeling + Phacoemulsification and IOL implantation in phakic eyes	Baseline, 1, 3, 6 months	**FAZ area**, **FAZ perimeter**, FAZ AI, FD-300 on the retina slab, **VD in the SCP** and DCP in 5 regions (fovea, S, I, N, T)	**Predictive**: D-value BCVA (degree of visual improvement postoperatively) positively correlated with foveal VD in SCP and negatively correlated with FAZ area and FAZ perimeter
Frisina et al., 2023 [42]	Prospective	40	Fellow healthy eye	PPV 27G with ERM and ILM peeling (all eyes were pseudophakic)	Baseline, 1, 3, 6 months	**FAZ area in the SCP**, VAD, VLF, VD index in the SCP and DCP, CC flow	**6 months**: Inverse correlation between FAZ area and BCVA
Kim et al., 2023 [43]	Retrospective observational	28	Fellow healthy eye	PPV 25G with ERM and ILM peeling + Gas C3F8 tamponade + Phacoemulsification and IOL implantation in phakic eyes	Baseline, minimum 6 months	**FAZ area**, **FAZ perimeter** and FAZ circularity in the SCP	**Baseline**: BCVA negatively correlated with FAZ area and FAZ perimeter **Predictive**: BCVA improvement and postoperative final BCVA was positively correlated with baseline FAZ area and FAZ perimeter
Liao et al., 2020 [44]	Retrospective	38	Fellow healthy eye	PPV 27G with ERM and ILM peeling	Baseline, 6 months	**FAZ area**, VD in the SCP and DCP	**6 months**: FAZ area negatively correlated with BCVA (univariate logistic analysis)
Yoshida et al., 2020 [45]	Retrospective	36	Fellow healthy eye	PPV 25G or 27G with ERM and ILM peeling ± Fluid/gas exchange in case of retinal breaks + Phacoemulsification and IOL implantation in phakic eyes	Baseline, 6 months	**FAZ area ratio in the SCP (interocular ratio)**	**Baseline**: FAZ area ratio negatively correlated with BCVA **Predictive**: FAZ area ratio negatively correlated with changes in the ETDRS letter score
Mastrogiuseppe et al., 2025 [46]	Prospective	57	Fellow healthy eye	PPV 25G with ERM and ILM peeling + Phacoemulsification and IOL implantation in phakic eyes	Baseline, 1 week, 1, 3, 6, 12 months	ONH: **Whole VD of RPC**, Inside-disc RPC VD, Peripapillary RPC VD Macula: FAZ area, FAZ perimeter, FD, **VD in the SCP** and DCP **of the whole macular region** (3 × 3 mm^2^), fovea, parafovea, flow area in the outer retina and CC	**Predictive**: Predictors of ΔBCVA in the multivariate linear regression analysis: wsVD, wRPC (higher wRPC VD and lower wsVD at baseline were considered as predictive factors for ΔBCVA)
Kim et al., 2024 [47]	Retrospective observational	28	Fellow healthy eye	PPV 25G with ERM and ILM peeling ± Phacoemulsification and IOL implantation (27)	Baseline and at least 6 months	**FAZ area**, **FAZ perimeter**, **FAZ circularity** in the SCP, **CC perfusion** measured at baseline only	**Baseline**: FAZ area negatively correlated with BCVA **Predictive**: Baseline FAZ area and perimeter positively correlated with BCVA improvement; baseline FAZ circularity negatively correlated with postoperative BCVA; baseline CCP positively correlated with BCVA improvement and postoperative BCVA, each confirmed by multivariate regression analysis
Lin et al., 2020 [48]	Retrospective	85	None	PPV 25G with ERM and ILM peeling ± Phacoemulsification and IOL implantation (4)	Baseline, 1 month, repeated every 1 or 2 months (minimum 1 year follow-up required)	FAZ area in the SCP and DCP, VD in the SCP and DCP in the parafoveal region	None identified
Li et al., 2025 [49]	Retrospective observational	46	Fellow healthy eye	PPV 23G with ERM and ILM peeling + Gas/ liquid exchange + Phacoemulsification and IOL implantation in case of cataract or refractive error	Baseline, 1, 3 months	**FAZ area**, FAZ perimeter, FD-300, **VT**, **VD in the SCP and DCP in the fovea** and parafovea	**Predictive**: BCVA-d correlated positively with baseline foveal VD in the SCP and VT, and negatively with FAZ area and foveal VD in the DCP; BCVA at 3-month correlated positively with baseline foveal VD in the SCP and VT, and negatively with FAZ area and foveal VD in the DCP
Rommel et al., 2020 [50]	Prospective, observational	63	Fellow healthy eye	PPV 23G with ERM and ILM peeling + Fluid/air exchange + Phacoemulsification and IOL implantation in phakic eyes	Baseline, 3 months	Full retinal perfusion, CC perfusion, **Sattler’s layer perfusion**, Haller’s layer perfusion	**Predictive**: Higher baseline Sattler’s layer perfusion predicts better postoperative BCVA, a finding confirmed in the multiple regression analysis
Mavi Yildiz et al., 2021 [51]	Retrospective	112 (64 performed baseline FAZ assessment)	Fellow healthy eye	PPV 27G with ERM and ILM peeling + Phacoemulsification and IOL implantation in case of cataract (10)	Baseline, 6, 12 months	FAZ in the SCP	None identified
Zhan et al., 2025 [52]	Retrospective	30	28 healthy eyes	PPV 25G with ERM and ILM peeling + Phacoemulsification and IOL implantation in patients over 55 years of age with mild cataract	Baseline, 3 months	VD, PA, **PC in the SCP** and DCP	**3 months**: BCVA correlated with PC in the 6 × 6 mm^2^ SCP region **Predictive**: Higher baseline PC in the SCP in both 3 × 3 mm^2^ and 6 × 6 mm^2^ regions was associated with greater RS improvement; postoperative RS positively correlated with baseline PC in the SCP in the 3 × 3 mm^2^ region, a finding confirmed in the multiple linear regression analysis
Baba et al., 2018 [53]	Retrospective	17	Fellow healthy eye	PPV 25G or 27G with ERM and ILM peeling + Phacoemulsification and IOL implantation in phakic eyes	Baseline, 3, 6, 12 months	**FAZ in the SCP**	**3 and 6 months**: FAZ area inversely correlated with the RS

Biomarkers in bold indicate statistically significant (*p* value < 0.05) associations with functional outcomes as reported in each study. AI—acircularity index, BCVA—best-corrected visual acuity, BD—direct vessel branching point distance, CC—choriocapillaris, CCP—choriocapillaris plexus, CH—choroid, DCP—deep capillary plexus, ERM—epiretinal membrane, FAZ—foveal avascular zone, FBP—foveal branching point, FD-300—vessel density within a 300 μm wide region around the FAZ, FD—fractal dimension, FVD—foveal vessel density, I—inferior, ILM—internal limiting membrane, IOL—intraocular lens, iERM—idiopathic epiretinal membrane, MS—macular sensitivity, MVR—macular vessel density ratio, N—nasal, OCTA—optical coherence tomography angiography, OCP—outer capillary plexus, OCT—optical coherence tomography, ONH—optic nerve head, PA—perfusion area, PC—perfusion capacity, PD—perfusion density, PPV—pars plana vitrectomy, PRVD—parafoveal vessel density, RPC—radial peripapillary capillaries, RS—retinal sensitivity, RVTI—retinal vascular tortuosity index, S—superior, SCP—superficial capillary plexus, T—temporal, VAD—vessel area density, VD—vessel density, VL—vessel length, VLD—vessel length density, VLF—vessel length fraction, VPD—vascular perfusion density, VT—vessel tortuosity.

**Table 3:** OCTA metrics definitions.

OCTA Parameter (Abbreviation)	Unit	Definition
Foveal avascular zone (FAZ) area	mm^2^	Measured area of avascularity in the foveal region circumscribed by the retinal vascular complexes
FAZ perimeter	mm	Length of the perimeter of the FAZ
FAZ circularity		A metric ranging from 0 to 1 that quantifies how closely the FAZ shape approximates a perfect circle, with 1 indicating perfect circularity
Acircularity index (AI)	%	A metric that quantifies the deviation of the FAZ from a perfect circle by comparing its measured perimeter to that of a circle with the same area, where 1 represents a perfect circle and higher values indicate greater distortion
Vessel/vascular density (VD) or Vessel area density (VAD)	%	The percentage of the scanned area occupied by blood vessels, calculated as the ratio of pixels representing vasculature to the total number of pixels in the scan
Average vessel length (VL)	mm	Lengths of all identified vessel segments along the centerline of the vessel
Vessel length density (VLD) or Vessel skeleton density (VSD) or Vessel length fraction (VLF)	%	Ratio of the total length of blood vessels to the total scanned area, with each vessel represented as a single-pixel-width line along its centerline
Foveal VD 300 (FD-300)	%	Vessel density within a 300 µm wide annulus surrounding the FAZ, calculated as the percentage of the area occupied by vessels within this rim
Flow area	mm^2^	The area or intensity of flow signal within a predefined region of interest
Vessel tortuosity (VT)		A metric that quantifies the curvature of vessels in the OCTA scan, calculated as the ratio of the segment length along the vessel centerline to the straight-line distance between its endpoints
Fractal dimension		A metric that quantifies the geometric complexity of the retinal vascular network, reflecting how vessels branch and fill space across different scales; higher values indicate a more complex and dense branching pattern

**Table 4:** Types of OCTA devices employed in the included studies.

OCTA Device	Studies (*n*; References)
AngioVue Imaging System (RTVue XR Avanti, Optovue Inc., Fremont, CA, USA)	24 studies; [9,12,14,15,16,17,18,23,25,26,27,28,30,31,32,34,35,36,41,44,46,48,49,53]
Carl Zeiss Meditec Inc. (Dublin, CA, USA)	6 studies; [19,22,39,40,45,50]
DRI OCT Triton (Topcon Corporation, Tokyo, Japan)	6 studies; [20,21,29,37,43,47]
NIDEK OCTA systems (NIDEK Co., Ltd., Gamagori, Japan)	5 studies; [13,24,33,38,42]
VG200 (S Vision Imaging, Luoyang, China)	1 study; [52]
Spectralis HRA + OCT (Heidelberg Engineering, Heidelberg, Germany) with integrated OCTA	1 study; [51]

## Data Availability

Not applicable.

## Supplementary Materials

The following supporting information can be downloaded at: https://www.mdpi.com/article/10.3390/diagnostics15202596/s1, Table S1. Risk of bias assessment.

## Abbreviations

The following abbreviations are used in this manuscript:

ERM	Epiretinal Membrane
iERM	Idiopathic Epiretinal Membrane
PPV	Pars Plana Vitrectomy
OCT	Optical Coherence Tomography
OCTA	Optical Coherence Tomography Angiography
VA	Visual Acuity
FAZ	Foveal Avascular Zone
AI	Acircularity Index
ILM	Internal Limiting Membrane
VD	Vessel Density
VL	Vessel Length
VLD	Vessel Length Density
FD-300	Foveal Vessel Density 300
VT	Vessel Tortuosity
SCP	Superficial Capillary Plexus
DCP	Deep Capillary Plexus
BCVA	Best-Corrected Visual Acuity
IPL	Inner Plexiform Layer
OPL	Outer Plexiform Layer
INL	Inner Nuclear Layer
EZ	Ellipsoid Zone
RS	Retinal Sensitivity
MS	Macular Sensitivity
CC	Choriocapillaris
CH	Choroid
