# Supplementary material for: OCTA Biomarkers Underlying Structure–Function Correlations in Idiopathic Epiretinal Membrane: A Systematic Review
Source: Diagnostics (Basel). 2025 Oct 15;15(20):2596. doi: 10.3390/diagnostics15202596 (PMC12562888; doi:10.3390/diagnostics15202596)
Supplement: Supplementary file 1 [file diagnostics-15-02596-s001.zip › diagnostics-3906855-supplementary.pdf]

Table S1. Risk of Bias Assessment

	Research question	Study population	Participation rate	Inclusion criteria	Sample size	Exposure prior to outcome	Sufficient timeframe	Different levels of exposure	Exposure measures	Multiple exposure	Outcome measures	Blinding of outcome	Loss to follow-up	Statistical analysis	Overall quality
Kim et al., 2018	Yes	Yes	Yes	Yes	No	No	Yes	No	Yes	No	Yes	NR	NA	No	Fair
Bacherini et al., 2021	Yes	Yes	CD	Yes	No	Yes	Yes	Yes	Yes	Yes	Yes	NR	CD	No	Fair
Zhang et. al., 2024	Yes	Yes	Yes	Yes	No	Yes	Yes	Yes	Yes	Yes	Yes	NR	NA	No	Fair
Isik-Ericek et. al., 2021	Yes	Yes	CD	Yes	No	Yes	Yes	Yes	Yes	Yes	Yes	NR	NR	No	Fair
Shen et. al., 2023	Yes	Yes	CD	Yes	No	Yes	Yes	Yes	Yes	Yes	Yes	NR	CD	Yes	Good
Okawa et. al., 2019	Yes	Yes	CD	Yes	No	Yes	No	Yes	Yes	Yes	Yes	NR	No	No	Poor
Feng et. al., 2021	Yes	Yes	Yes	Yes	No	Yes	Yes	Yes	Yes	Yes	Yes	NR	NA	Yes	Fair
Miyazawa et. Al., 2022	Yes	Yes	CD	Yes	No	Yes	Yes	Yes	Yes	Yes	Yes	NR	CD	Yes	Fair
Yuce et. al., 2021	Yes	Yes	CD	Yes	No	Yes	Yes	Yes	Yes	Yes	Yes	NR	NA	No	Fair
Bae and Ryoo, 2022	Yes	Yes	No	Yes	No	Yes	Yes	Yes	Yes	Yes	Yes	NR	NA	Yes	Fair
D'Aloisio et. al., 2021	Yes	Yes	CD	Yes	No	Yes	Yes	Yes	Yes	Yes	Yes	NR	CD	No	Fair
Li et. al., 2019	Yes	Yes	CD	Yes	No	Yes	Yes	Yes	Yes	Yes	Yes	NR	NR	Yes	Fair
Nicolai et. al., 2024	Yes	Yes	CD	Yes	No	Yes	Yes	Yes	Yes	No	Yes	NR	NR	No	Poor
Li et. al., 2023	Yes	Yes	No	Yes	No	Yes	Yes	Yes	Yes	No	Yes	NR	CD	Yes	Fair
Chen et. al., 2019	Yes	Yes	CD	Yes	No	Yes	Yes	Yes	Yes	Yes	Yes	NR	CD	No	Fair
Xu et. al., 2021	Yes	Yes	Yes	Yes	No	Yes	Yes	Yes	Yes	Yes	Yes	NR	Yes	No	Fair
Yanık et. al., 2023	Yes	Yes	Yes	Yes	No	Yes	No	Yes	Yes	Yes	Yes	NR	CD	No	Fair
Caretti et. al., 2024	Yes	Yes	CD	Yes	No	Yes	Yes	Yes	Yes	Yes	Yes	NR	CD	No	Fair
Kim and Park, 2021	Yes	Yes	CD	Yes	No	Yes	Yes	Yes	Yes	Yes	Yes	NR	NA	Yes	Fair
Ersoz et. al., 2021	Yes	Yes	CD	Yes	No	Yes	Yes	Yes	Yes	Yes	Yes	NR	NA	Yes	Good
Mao et. al., 2021	Yes	Yes	Yes	Yes	No	Yes	Yes	Yes	Yes	Yes	Yes	NR	Yes	No	Fair
Chatzistergiou et. al., 2021	Yes	Yes	CD	Yes	No	Yes	Yes	Yes	Yes	Yes	Yes	NR	NA	Yes	Fair
Henry et. al., 2024	Yes	Yes	CD	Yes	No	Yes	Yes	Yes	Yes	Yes	Yes	NR	NA	Yes	Fair
Xu et. al., 2024	Yes	Yes	Yes	Yes	No	Yes	Yes	Yes	Yes	Yes	Yes	NR	CD	Yes	Good
Hondur and Aribas, 2024	Yes	Yes	CD	Yes	No	Yes	Yes	Yes	Yes	Yes	Yes	NR	NA	Yes	Good
Wang et. al., 2023	Yes	Yes	CD	Yes	No	Yes	Yes	Yes	Yes	Yes	Yes	NR	NR	Yes	Good
Told et. al., 2020	Yes	Yes	Yes	Yes	No	Yes	Yes	Yes	Yes	Yes	Yes	NR	Yes	No	Fair
Osada et. al., 2020	Yes	Yes	CD	Yes	No	Yes	Yes	Yes	Yes	Yes	Yes	NR	CD	Yes	Good
Hirata et. al., 2019	Yes	Yes	CD	Yes	No	Yes	Yes	Yes	Yes	Yes	Yes	NR	NR	Yes	Good
Honzawa et. al., 2023	Yes	Yes	CD	Yes	No	Yes	Yes	Yes	Yes	No	Yes	NR	CD	No	Fair
Mao et. al., 2020	Yes	Yes	CD	Yes	No	Yes	Yes	Yes	Yes	Yes	Yes	NR	CD	No	Fair
Frisina et. al., 2023	Yes	Yes	CD	Yes	Yes	Yes	Yes	Yes	Yes	Yes	Yes	NR	CD	No	Fair
Kim et. al., 2023	Yes	Yes	CD	Yes	No	Yes	Yes	Yes	Yes	Yes	Yes	NR	CD	No	Fair
Liao et. Al., 2020	Yes	Yes	CD	Yes	No	Yes	Yes	Yes	Yes	Yes	Yes	NR	NA	No	Fair
Yoshida et. al., 2020	Yes	Yes	CD	Yes	No	Yes	Yes	Yes	Yes	Yes	Yes	NR	NA	No	Fair
Mastrogiuseppe et. al., 2025	Yes	Yes	Yes	Yes	Yes	Yes	Yes	Yes	Yes	Yes	Yes	NR	NR	Yes	Good
Kim et. al., 2024	Yes	Yes	CD	Yes	No	Yes	Yes	Yes	Yes	Yes	Yes	NR	CD	Yes	Fair
Lin et. al., 2020	Yes	Yes	CD	Yes	No	Yes	Yes	Yes	Yes	Yes	Yes	NR	NA	Yes	Fair
Li et. al., 2025	Yes	Yes	CD	Yes	No	Yes	Yes	Yes	Yes	Yes	Yes	NR	CD	No	Fair
Rommel et. al., 2020	Yes	Yes	CD	Yes	No	Yes	Yes	Yes	Yes	Yes	Yes	NR	NR	Yes	Fair
Mavi Yildiz et. al., 2021	Yes	Yes	Yes	Yes	No	Yes	Yes	No	Yes	Yes	Yes	NR	NA	Yes	Fair
Zhan et. al., 2025	Yes	Yes	CD	Yes	Yes	Yes	Yes	Yes	Yes	Yes	Yes	NR	NA	Yes	Good
Baba et. al., 2018	Yes	Yes	CD	Yes	No	Yes	Yes	No	CD	Yes	Yes	NR	CD	No	Poor
